# Event-related brain dynamics during mind wandering in attention-deficit/hyperactivity disorder: An experience-sampling approach

**DOI:** 10.1016/j.nicl.2022.103068

**Published:** 2022-06-01

**Authors:** Natali Bozhilova, Jonna Kuntsi, Katya Rubia, Philip Asherson, Giorgia Michelini

**Affiliations:** a Social, Genetic and Developmental Psychiatry Centre, Institute of Psychiatry, Psychology and Neuroscience, King’s College London, De Crespigny Park, London SE5 8AF, UK; b School of Psychology, University of Surrey, Guildford, UK; c Department of Child and Adolescent Psychiatry, Institute of Psychiatry, Psychology and Neuroscience, King’s College University London, De Crespigny Park, London SE5 8AF, UK; d Semel Institute for Neuroscience & Human Behavior, University of California Los Angeles, 760 Westwood Plaza, Los Angeles, CA 90024, United States; e Department of Biological & Experimental Psychology, Queen Mary University of London, G. E. Fogg Building, Mile End Road, London E1 4NS, UK

**Keywords:** Brain oscillations, EEG, Mind wandering, ADHD, Adult, Working memory, Sustained attention

## Abstract

Adults with attention-deficit/hyperactivity disorder (ADHD) report increased spontaneous mind wandering (MW) compared to control adults. Since MW is associated with ADHD severity and functional impairment, elucidating the brain mechanisms underlying MW may inform new interventions targeting MW and point to neural markers to monitor their efficacy. Population-based electroencephalographic (EEG) studies suggest that weaker event-related decreases in occipital alpha power characterise periods of MW, but no study has examined event-related brain oscillations during MW in individuals with ADHD. Using an experience-sampling method, we compared adults with ADHD (N = 23) and controls (N = 25) on event-related EEG measures of power modulations and phase consistency during two tasks with high and low demands on working memory and sustained attention, and during periods of MW and task focus. Compared to controls, individuals with ADHD showed weaker alpha power decreases during high working memory demands and across sustained attention demands, weaker theta power increases and phase consistency across working memory demands and during low sustained attention demands, and weaker beta power decreases during low working memory demands. These EEG patterns suggest broadly deficient attentional and motor response processes in ADHD. During MW episodes, adults with ADHD showed weaker alpha power decreases in the sustained attention task and lower theta phase consistency in the working memory task compared to controls. These findings suggest that atypical EEG patterns thought to reflect reduced inhibition of task-irrelevant processes and inconsistent stimulus processing underlie increased MW in adults with ADHD and may be useful for future real-time monitoring of treatment effects.

## Highlights

• ADHD was associated with atypical modulation of EEG power across varying levels of cognitive demand.
• This atypical modulation was most pronounced during MW episodes and under sustained attention conditions.
• These EEG patterns broadly indicate reduced inhibition of task-relevant processes and poor motor control.

## 1 Introduction

Attention-deficit/hyperactivity disorder (ADHD) is an impairing neurodevelopmental disorder, defined by developmentally inappropriate levels of inattention and hyperactivity/impulsivity (APA, 2013). ADHD affects 7% of children and 2.8% of adults worldwide (Faraone et al., 2021). Across the lifespan, individuals with ADHD commonly experience excessive spontaneous mind wandering (MW) (Asherson, 2005), which reflects involuntary shift of attention away from the current task (Mowlem et al., 2016; Frick, Asherson, & Brocki, 2020). These findings led to the proposition of a new MW perspective on ADHD, which states that atypical regulation of neural activity underlying MW might explain ADHD symptoms and related impairments in cognitive performance (Bozhilova, Michelini, Kuntsi, & Asherson, 2018). Thus, elucidating the neural mechanisms underlying excessive MW in ADHD may inform the development of new treatments to alleviate attentional difficulties, as well as objective neural markers for real-time monitoring treatment effects. However, there is still very limited data on the neural mechanisms underlying periods of MW in individuals with ADHD.

Functional magnetic resonance imaging (fMRI) studies have identified the default mode network (DMN) as a neural correlate of MW (Christoff et al., 2009, Mason et al., 2007, Fox et al., 2015). However, MW is a transient process that may be more adequately captured by the advanced temporal resolution of EEG (Callard et al., 2013). Time-frequency analyses of EEG signals can examine transient dynamics in event-related brain oscillations, thought to be implicated in functional coupling within and between neural networks (Kirschner et al., 2012, Delorme and Makeig, 2004). Experience-sampling studies with undergraduate students, asking them to self-report episodes of MW (i.e., self-caught MW) or answer whether they were on– or off-task (i.e., probe-caught MW), found that MW is associated with a weaker event-related decrease in alpha and beta power (Braboszcz and Delorme, 2011, Van Son et al., 2019, Baird et al., 2014). These EEG patterns have been interpreted as reduced inhibition of task-irrelevant information and motor response processes, respectively. Studies of professional meditators also showed weaker frontal theta and somatosensory alpha power during self-caught MW compared to meditation (Brandmeyer, & Delorme, 2018), suggesting that MW is accompanied by neural processes reflecting reduced attention allocation and somatosensory processing. Other studies on community samples further linked probe-caught MW to reduced theta phase consistency (Baldwin et al., 2017, Arnau et al., 2020), thought to reflect neural variability in stimulus processing (Groom et al., 2010). Overall, although few EEG studies of MW are available, weaker alpha power decrease emerges as one of the most replicated correlates of MW (Jin, Borst, & van Vugt, 2019).

Recent reviews have found that weaker event-related alpha decreases during attentional and working memory tasks are reliably observed in individuals with ADHD relative to controls (Lenartowicz et al., 2018, Michelini et al., 2022). These findings suggest that EEG markers thought to reflect reduced inhibition of task-irrelevant information robustly characterise ADHD. One study also found that individuals with ADHD, unlike controls, did not show improvements in alpha decrease from slow, unrewarded conditions to faster and incentivised conditions (Michelini et al., 2018), suggesting poor regulation of alpha activity with changing task demands. Further evidence points to weaker beta power decreases and shorter beta power increases in individuals with ADHD compared to controls during basic somatosensory processing, respectively indicating impaired motor response execution and inhibition (Dockstader et al., 2008, Hasler et al., 2016). Additionally, studies on ADHD samples have reported reduced event-related theta increase and phase consistency during cognitive tasks (Groom et al., 2010, Michelini et al., 2018, Juurmaa et al., 2020, Khoshnoud et al., 2018, Michelini et al., 2022). Of note, these alterations in theta activity were recently linked to task increased variability in reaction times and developmental persistence of ADHD into adulthood (Vainieri et al., 2020).

We have recently found that event-related power modulations distinguishing adults with ADHD from controls (i.e., weaker alpha and beta decrease during response inhibition and weaker theta increase during response execution) were associated with self-reported spontaneous MW (Bozhilova et al., 2021a). These findings support the hypothesis that ADHD and spontaneous MW may share the same neural deficits (Bozhilova et al., 2018). However, this study measured MW with a self-reported questionnaire, rather than taking an experience-sampling approach for distinguishing between periods of MW and task focus during a cognitive task in real time. Using cognitive performance and event-related potential (ERP) measures in the sample used for the current study, we recently found that adults without ADHD maintained consistent task focus with increasing demand on both working memory and sustained attention (i.e., context regulation) (Bozhilova et al., 2020, Bozhilova et al., 2021b, Bozhilova et al., 2021a, Bozhilova et al., 2021b). Instead, adults with ADHD showed deficient context regulation of MW (i.e., increased MW frequency) during high cognitive demand on sustained attention, but not on working memory (Bozhilova et al., 2020, Bozhilova et al., 2021a, Bozhilova et al., 2021b). With regards to our previous ERP findings, we found that individuals with ADHD showed significantly reduced P1 amplitudes (reflecting early sensory processes) relative to controls during periods of task focus, but no differences during MW episodes. Conversely, P3 amplitudes (reflecting attention allocation) were lower in those with ADHD than controls during MW but not during task focus (Bozhilova et al., 2021a, Bozhilova et al., 2021b). Given the strong association of MW with ADHD symptomatology and associated daily life difficulties (Bozhilova et al., 2018), identifying the neural correlates of real-time MW levels may suggest new ways to monitor treatment effects in individuals with ADHD, with the potential to improve future treatment practices.

To further elucidate the neural mechanisms of MW, we carried out a new in-depth analysis of the data presented in our previous publication (Bozhilova et al., 2021a, Bozhilova et al., 2021b), using event-related brain oscillatory analysis. This time–frequency approach can investigate both spectral and time-related aspects of the EEG data and thus provides richer information on the neural bases of fast-changing cognitive processes compared to cognitive performance and ERP measures (Michelini et al., 2022). Specifically, we focused on event-related modulations of alpha, beta and theta power, and theta phase consistency, which are markers of attentional and motor response processes previously associated with MW in community samples and with ADHD-control differences (Braboszcz and Delorme, 2011, Van Son et al., 2019, Baird et al., 2014, Groom et al., 2010, Michelini et al., 2022). Our first aim was to compare ADHD and control groups on event-related oscillatory measures during two tasks with high and low demands on working memory (1-back vs. 0-back) and sustained attention (varying stimulus onset delays of 1 s, 2 s, 5 s and 8 s), which elicit varying levels of MW (Analysis 1). We hypothesised a-priori that individuals with ADHD would show weaker event-related alpha, beta and theta power modulations and lower theta phase consistency than controls. Based on our previous findings of task-related changes in EEG activity and MW in adults with ADHD, but not in controls (Bozhilova et al., 2021a, Bozhilova et al., 2020), we also predicted that only the ADHD group would show a within-group reduction in EEG power modulations and phase consistency from the 0-back condition to the 1-back condition. Finally, we hypothesised that MW frequency during these tasks would statistically account for these effects, suggesting a role of MW in the atypical EEG patterns displayed by the ADHD group. Our second aim was to examine event-related oscillatory activity during periods of MW and task focus in the two groups (Analysis 2). We predicted that the ADHD group would display atypical EEG patterns compared to controls during periods of MW, but not during task focus. This would suggest that atypical brain profiles in adults with ADHD are limited to periods of MW and that longer periods of task focus may potentially bring about EEG profiles more like controls. Finally, we hypothesised that controls, but not adults with ADHD, would show a within-group enhancement in EEG power modulations and phase consistency from MW to task focus, suggesting an effective neural adaptation from processing of task-irrelevant information to processing of goal-directed information.

## 2 Methods

The sample consisted of 23 participants with ADHD and 25 controls with good quality EEG data (‘*EEG analyses/data screening’ section*) from our initial sample of 27 adults with ADHD and 29 controls. Recruitment centres for adults with ADHD were the South London and Maudsley NHS Trust, the Barnet, Enfield and Haringey Mental Health Trust adult ADHD clinics, online platforms, UKAAN (the UK Adult ADHD Network) and mental health professionals. Adults without ADHD (i.e., one or no ADHD symptoms based on the clinical assessments during this study), and no prior diagnosis for mental health conditions were recruited via online recruitment platforms. Exclusion criteria for both groups included a current or past diagnosis of major physical illness (e.g., neurological problems, head injury), severe mental health difficulties (e.g., psychosis, schizophrenia, bipolar disorder, antisocial personality disorder), current or past substance abuse (defined as more than 8 units of alcohol for males or 6 units for females of alcohol per day, or recreational drug use more than twice weekly), or an IQ below 80.

All adults with ADHD provided a clinical record of formal ADHD diagnosis and met both DSM-IV and DSM-V ADHD criteria, as confirmed with the Diagnostic Interview for ADHD (DIVA 2.0) during clinical assessments ADHD (Kooij, 2012). Twelve adults with ADHD were on continuous treatment with stimulants and two were treated with atomoxetine. Seven adults with ADHD were also on medication for anxiety and/or depression. There were no between-group differences regarding age, sex, and IQ (Table 1). This study has been reviewed and approved by the Newcastle North Tyneside 1 NHS Ethics Committee (17/NE/0188). All participants provided informed consent prior to participation.

**Table 1:** Comparisons between ADHD and control group on demographic characteristics.

	ADHD (N = 23)	Controls (N = 25)	Group comparison	
	Mean ± SD	Mean ± SD	d	p
Age (years)	36.73 ± 8.67	31.80 ± 11.42	0.47	0.113
IQ	111.50 ± 13.25	114.28 ± 16.72	0.18	0.528
MW frequency	0.57 ± 0.22	0.15 ± 0.14	2.16	0.001*

	Males: Females	Males: Females	Chi^2^	p

Gender	13:10	12:13	0.47	0.521

Abbreviations: ADHD- Attention-deficit/hyperactivity disorder, IQ- Intelligent Quotient from the Wechsler Abbreviated Scale of Intelligence, WASI-II.

Notes: The total MW frequency was calculated using the total number of MW episodes across tasks divided by the total number of all episodes (task focus and MW). *p < 0.05.

### 2.1 Procedure

The testing session for all participants lasted approximately 3–4 h and included a clinical interview for ADHD (DIVA 2.0), IQ testing and self-report measures, and two computerised tasks with simultaneous EEG recordings preceded by a practice session for each task (Bozhilova et al., 2021a, Bozhilova et al., 2021b). Participants were asked not to smoke, consume caffeinated/alcoholic drinks, and take non-illicit substances on the day of the testing. Participants with ADHD were also asked to discontinue their treatment with stimulants for 48 h before the assessment, as is regular practice in cognitive/EEG studies of ADHD samples (Michelini et al., 2016). On the testing day, all participants provided a written record that they had complied with these requests.

## 3 Cognitive tasks

### 3.1 Mind wandering task (Konishi et al., 2015)

This task consists of a 0-back and 1-back condition. The 0-back is a choice reaction condition designed to capture alertness levels and motor activity. By contrast, the 1-back is a working memory condition, which aims to assess visual working memory. In the 0-back condition, a sequence of black shapes (separated by a blue line) is presented to the participant in the middle of the computer screen. The participant is instructed to observe these shapes before a blue target appears (a small shape with two bigger shapes on each side). Once the target is presented, the participant had to use the left or the right arrow to select the location of the bigger shape matching the location of the middle target shape. In the 1-back condition, the same sequence of black shapes (separated by a red line) was occasionally paired with two red question marks (‘?’) with a small red shape (target) between the question marks. Upon presentation of the question marks, a manual response is required to indicate the location (left or right) of the target shape in the previous trial. The colored question marks appeared randomly, which required encoding and retaining in memory the location of each black shape in the previous trial (Supplementary Fig. 1).

These two task conditions occurred in a counterbalanced fashion. During each trial, 2 to 6 non-targets appeared before the target. The duration of each non-target was 1 to 3 s with increments of 0.1 s in each trial (the maximum duration was 3 s). The task had a total of 128 targets (64 in each condition) and 580 non-targets (290 in each condition). The duration of each target was 4 s, which allowed a 4 s time window for a response before the trial ended. A fixation cross was included before and after all task stimuli, which lasted 2 to 4 s with increments of 0.1 s.

The total number of trials in each block was 8 for each condition. The total number of blocks was also 8, and the duration of each block varied from 40 s to 120 s. The end of each block included two on-screen messages “STAY” or “SWITCH”, indicating to the participants that they were about to either remain the same condition or enter the other condition. The duration of both messages “SWITCH” and “STAY” was 5 s. The task lasted approximately 30 min split into two 15-min sessions.

### 3.2 Sustained attention task (SAT) (Christakou et al., 2013)

For this study we used a modified version of the SAT (Christakou et al., 2013). The original task measures vigilance by introducing 3 levels (2 s, 5 s, 8 s) of a progressively increasing load on sustained attention (Supplementary Fig. 1). An immediate response is required to the appearance of a millisecond counter (i.e., black digits). The participants can respond with a right button click within 1 s. As soon as the response is given, the next stimulus appears. The target duration is 1 s in the absence of a response. The counter occurred either after predictable intervals of 1 s, in series of 3 to 5 stimuli (520 in total, 260 in each session), or after unpredictable delays of 2, 5 or 8 s (52 in total, 26 each in each session), which were also pseudo-randomly allocated to blocks of 3 to 5 trials of 1 s. The unpredictable delays place a varying level of demand on attentional processes (lower for 2 s and higher for 8 s), whereas the predictable delays place greater demand on sensorimotor processes (Christakou et al., 2013). This task also lasted approximately 30 min split into two 15-min sessions (Supplementary Fig. 1).

### 3.3 MW probes

To capture MW, we used an experience-sampling approach with thought probes (15 per session, 30 in total) at approximately 1-minute intervals. The targets in the MWT and the stimulus following the unpredictable delays in the SAT were occasionally substituted by the MW probes. Our version of the SAT included 26 delays per session (78 in total) in contrast to 20 delays (60 in total) in the previous version of the task (Christakou et al., 2013). The inclusion of the extra delays (36 in total) allowed us to add more thought probes (30 in total), ensuring a consistent number of delays between our and the previous version of the SAT. The MW probes had the following text “*Where was your attention just before this probe?*” with two response options “*On task*” and “*Off task*”. If the participants responded, “*Off task*”, an additional question appeared “*Were you aware of your attention drifting away from the task?”* with two response options *“Aware”* and “*Unaware”.* To capture episodes of MW and task focus, we used the 15 s-time window prior to each probe, consistent with our previous ERP study (Bozhilova et al., 2021a, Bozhilova et al., 2021b) and previous MW approaches (Baird et al., 2014; Braboszcz et al., 2011; Kirschner et al., 2012).

### 3.4 EEG recoding and pre-processing

As described in our previous publication (Bozhilova et al., 2021a, Bozhilova et al., 2021b), we recorded the EEG data using a 62-channel DC-coupled recording system (extended 10–20 montage) (Brain Products, Gilching, Germany), a 500 Hz sampling rate, impedances under 10 kΩ, and FCz as the recording reference. The EEG recordings were imported and processed using EEGLAB (Delorme, & Makeig, 2004). We pre-processed the raw data using the following approach. We down sampled the data to 256 Hz, re-referenced to the average of all electrodes (turning FCz into an active channel), and used basic Finite impulse response (FIR) filters below 1 Hz and above 30 Hz. Prior to re-referencing, we removed flat channels and channels with extremely large artefacts and replaced their activity with topographic spline interpolation. An automatic algorithm also removed sections of data >200 μV. Ocular, muscle, and heart artefacts as well as line noise were corrected using independent component analysis (ICA) with the Adaptive Mixture ICA (AMICA) algorithm (Palmer et al., 2012), which is designed to remove the artefactual components and allow back-projection of all but those components. Following this ICA step, we carried out a visual inspection and manually removed residual artefacts.

### 3.5 Time-frequency analyses

For Analysis 1, we carried out separate EEG analyses on the working memory (1-back) and choice reaction (0-back) conditions of the MWT and on the delays (1 s, 2 s, 5 s, 8 s) of the SAT. This analysis did not include the trials preceding or containing MW probes to ensure consistency with previous studies using these tasks without thought probes. Instead, Analysis 2 specifically focused on the trials in the 15 s period preceding probes.

**Fig. 1:**
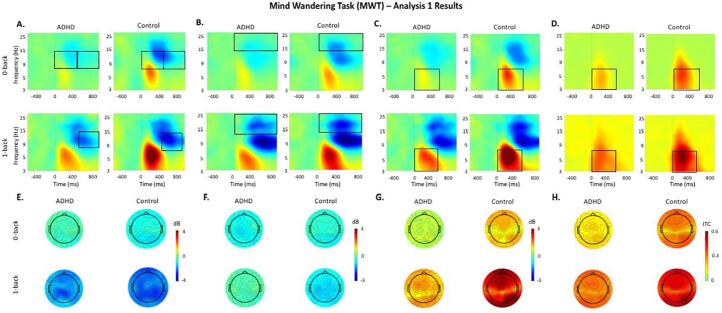
Event-related modulations during each task condition (0-back, 1-back) of the Mind Wandering Task (MWT) in the ADHD and control groups. Black boxes indicate significant group differences. The same scale limits are used across each time–frequency plot (A, B, C, D) and the corresponding topographic maps (E, F, G, H). A: Alpha (8–14 Hz) event-related perturbation (ERSP) at parieto-occipital regions. B: Beta (14–30 Hz) ERSP at centro-parietal regions. C: Theta (3–7 Hz) ERSP at fronto-central regions. D: Theta (3–7 Hz) inter-trial phase coherence (ITC) at fronto-central regions. E: Topographic maps for alpha ERSP in the 0–1000 ms window. F: Topographic maps for alpha ERSP in the 0–1000 ms window. G: Topographic maps for theta ERSP in the 0–500 ms window. H: Topographic maps for theta ITC in the 0–500 ms window.

**Fig. 2:**
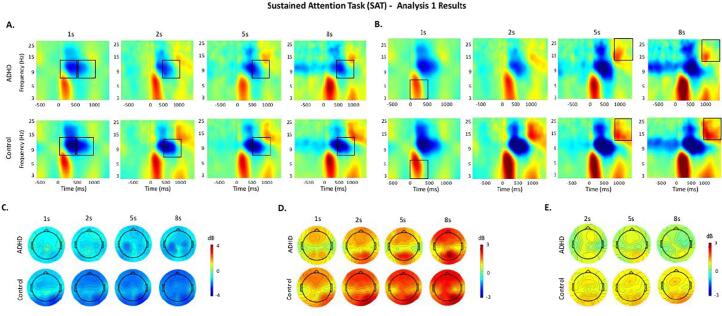
Event-related modulations during each delay (1 s, 2 s, 5 s, 8 s) of the Sustained Attention Task (SAT) in the ADHD and control groups. Black boxes indicate significant group differences. The same scale limits are used across each time–frequency plot (A, B) and the corresponding topographic maps (C, D, E). A: Alpha (8–14 Hz) event-related perturbation (ERSP) at parieto-occipital regions. B: Theta (3–7 Hz) and beta (14–30 Hz) ERSP at centro-parietal regions. C: Topographic maps for alpha ERSP in the 0–1000 ms window. D: Topographic maps for theta ERSP in the 0–500 ms window. E: Topographic maps for beta ERSP in the 750–1500 ms window. Note: topographic maps are not shown for the 1 s delay condition as beta increase was not measured in this condition due to the shorter inter-stimulus interval.

**Fig. 3:**
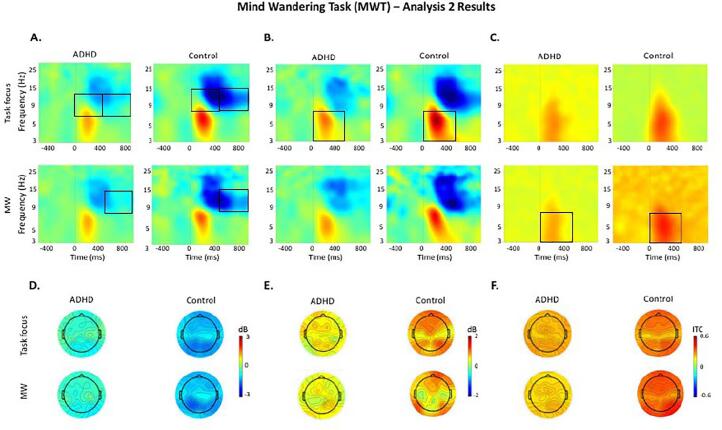
Event-related modulations during task focus and mind wandering (MW) in the mind wandering task (MWT) in the ADHD and control groups. Black boxes indicate significant group differences. The same scale limits are used across each time–frequency plot (A, B, C) and the corresponding topographic maps (D, E, F). A: Alpha (8–14 Hz) event-related spectral perturbation (ERSP) at occipital-parietal regions. B: Theta (3–7 Hz) ERSP at fronto-central regions. C: Theta (3–7 Hz) inter-trial phase coherence (ITC) at fronto-central regions. D: Topographic maps in the 0–1000 ms window for alpha ERSP. E: Topographic maps in the 0–500 ms window for theta ERSP. F: Topographic maps in the 0–500 ms window for theta ITC.

**Fig. 4:**
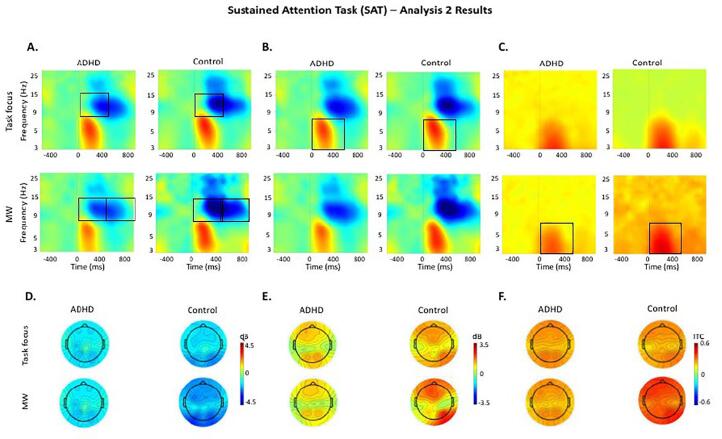
Event-related modulations during task focus and mind wandering (MW) in the sustained attention task (SAT) in the ADHD and control groups. The same scale limits are used across each time–frequency plot (A, B, C) and the corresponding topographic maps (D, E, F). A: Alpha (8–14 Hz) event-related spectral perturbation (ERSP) at occipital-parietal regions. B: Theta (3–7 Hz) ERSP at fronto-central regions. C: Theta (3–7 Hz) inter-trial phase coherence (ITC) at fronto-central regions. D: Topographic maps in the 0–1000 ms window for alpha ERSP. E: Topographic maps in the 0–500 ms window for theta ERSP. F: Topographic maps in the 0–500 ms window for theta ITC.

Time-frequency analyses were adopted to investigate changes in power and phase consistency related to task conditions (Analysis 1) and periods of MW and task focus (Analysis 2). Power changes were quantified as an event-related spectral perturbation (ERSP) index (Delorme and Makeig, 2004), using Morlet wavelet decomposition with linearly increasing number of cycles (frequency step of 0.80 Hz) from 3 cycles for the lowest frequency (3 Hz) to 25.6 cycles for the highest frequency (30 Hz). This approach optimises the trade-off between temporal resolution at lower frequencies and frequency resolution at higher frequencies, allowing for improved frequency resolution at higher frequencies. The average ERSP plots display decibel (dB) units of event-related increases (in red) and decreases (in blue) in the spectral power at a given frequency and latency with respects to pre-stimulus activity (Fig. 1, Fig. 2, Fig. 3, Fig. 4) from which frequency-specific ERSPs can be extracted. Phase consistency was measured as an inter-trial phase coherence (ITC) index calculated from the same Morlet wavelets. The ITC index shows the level of phase consistency of the evoked response across all trials at a given latency and frequency (Tallon-Baudry et al., 1996, Makeig et al., 2004, Delorme and Makeig, 2004). ITC values range from 0 (reflecting absence of phase consistency and highest phase variability across trials) to 1 (indicating perfect phase consistency and lowest phase variability). High phase consistency over trials is proposed to underlie stable neural processing of a stimulus, or phasic consistency in the neural response across trials (Makeig et al., 2004).

In the MTW, time–frequency analyses were applied for working memory/choice-reaction (1-back/0-back) conditions and periods of MW and task focus between −1500 to 1500 ms, normalized with respect to the mean log-power spectrum from a pre-stimulus period (baseline) between −500 to 0 ms. During the SAT, time–frequency analyses were applied between −1500 and 1500 ms for the 1 s condition, using a pre-stimulus baseline period between −500 to 0 ms; between −2500 and 2500 ms for the 2 s condition, using a baseline period between −1000 and 0 ms; between −5500 and 2500 ms for the 5 s condition, with a baseline period between −4000 and −3000 ms; and between 8500 ms and 2500 ms for the 8 s condition, with a baseline period between −7000 and −6000 ms. This different segmentation allowed us to ensure comparability of the baseline period across 2 s, 5 s, 8 s delays conditions in within-group comparisons, as the same 1000 ms period after presentation of the previous stimulus was used as a baseline for the ERSP/ITC indices across delays (for further details, see Supplementary Fig. 2). A shorter baseline (-500–0 ms) was used for the 1 s condition because a longer baseline (-1000–0 ms) would have captured the time of the response (Supplementary Fig. 2). Within-group comparisons only focused on the longer delays (2 s, 5 s, 8 s), which place varying demands on sustained attention, and not on the 1 s delay condition, which instead place high demand on sensorimotor function (Christakou et al, 2013). As such, the use of a shorter baseline in the 1 s condition did not introduce discrepancies between conditions. The between-group comparisons involved all four delays (1 s, 2 s, 5 s, 8 s).

The choice of time windows and scalp locations was based on the location and timing of maximal power changes in the relevant frequencies in previous time–frequency studies during similar tasks. These parameters were then confirmed based on maximal changes in the same frequencies in our data (Fig. 1, Fig. 2, Fig. 3, Fig. 4). Stimulus-locked ERSP in the theta (3–7 Hz) band was computed between 0 and 500 ms over fronto-central areas using the average of electrodes: FCz, Cz, C1, C2, FC1, FC2 in the MWT and over parietal regions (average of P3-P8, Pz POz, PO3-PO8) in the SAT (Groom et al., 2010, Michelini et al., 2018). Alpha (8–14 Hz) ERSP was measured between 0 and 500 ms and 500–1000 ms over parieto-occipital regions (average of Oz, O1, O2, P3-P8, POz, PO3-PO8) in both tasks (Bickel et al., 2012, Mazaheri and Picton, 2005). Beta (15–30 Hz) ERSP was extracted in the MWT over centro-parietal (average of C1-C4, CPz, CP1-CP4) between 0 and 1000 ms, and in the SAT over parietal regions (average PO3-PO7, POz, P3-P8) between 0 and 750 ms across all delays and between 750 and 1500 ms in the 2 s, 5, and 8 s delays (Bickel et al., 2012, Mazaheri and Picton, 2005). Analyses of this later time window captured a beta increase following response in the longer delays in this task and were not performed in the 1 s condition as the next stimulus appears after 1000 ms. ITC was measured over the same scalp regions used for theta ERSP between 0 and 500 ms only in the theta band, where greater phase consistency in response to the event was expected based on previous studies (Groom et al., 2010, Michelini et al., 2018).

Participants were included in the ERSP/ITC analyses if they had at least 20 artefact-free EEG segments in each condition or probe. This approach is in line with previous requirements of at least 20 artefact-free EEG segments to observe reliable neural effects (Rietdijk et al., 2014). For Analysis 1, 4 out of the original 27 individuals with ADHD and 4 out of 29 controls were excluded due to faulty data files or low data quality (e.g., extremely large artefacts). Analysis 1 thus included data from 23 individuals with ADHD and 25 controls. For Analysis 2*,* we further removed 4 additional controls and 2 individuals with ADHD due to the absence of MW and task focus episodes in the MWT, leaving data from 21 individuals with ADHD and 21 controls for this analysis. Seven controls did not have sufficient MW episodes (>3 episodes) in the SAT, leaving us with 23 individuals with ADHD and 18 controls for analysis. For more details on the average number of trials included in each ESRP/ITC measure, please refer to Supplementary Table 3.

## 4 Statistical analyses

In Analysis 1, we studied the effects of condition (1-back/0-back for MWT; 1 s/2s/5s/8s for SAT), group (ADHD/control) and condition-by-group interactions on each ESRP/ITC in repeated measures general linear models. For alpha and beta, different time windows were tested in a separate repeated measures general linear model. To understand the effect of MW frequency on the EEG variables, we then repeated the same analyses adding MW frequency as a covariate. In Analysis 2, we measured the effects of probe (MW/task focus), group (ADHD/control) and probe-by-group interactions on each EEG measure in repeated measures general linear models. In both analyses, for measures showing significant main effects of group and/or condition/probe, we carried out additional post-hoc analyses comparing groups in each condition/probe separately and comparing conditions in each group based on the following a-priori predictions (even in the absence of significant interactions, which our study may not be powered to detect):

*Prediction 1 (Analysis 1)*: Individuals with ADHD would show weaker event-related alpha, beta and theta power modulations and lower theta phase consistency than controls across task conditions.

*Prediction 2 (Analysis 1)*: Only the ADHD group would show a within-group reduction in EEG power modulations and phase consistency from the 0-back condition to the 1-back condition of the MWT.

*Prediction 3 (Analysis 2)*: In both tasks, the ADHD group would display atypical EEG patterns compared to controls during periods of MW, but not during task focus.

*Prediction 4 (Analysis 2)*: Controls, but not adults with ADHD, would show a within-group enhancement in EEG power modulations and phase consistency from MW to task focus.

All ERSP/ITC measures were normally distributed. A false discovery rate (FDR) threshold for between- and within-group effects separately in Analysis 1 and 2 was used to address multiple testing (Table 2). FDR significant p-values were p ≤ 0.014 for the between-group comparisons, and p ≤ 0.003 for the within-group comparisons in Analysis 1 and 2. The within- and between-group effects not surviving FDR correction and showing p < 0.05 are presented as trend-level effects that require further testing. Cohen’s d with correction for small sample sizes (n < 50) was generated for between-group and within-group comparisons (Lakens, 2013). We interpreted our findings considering both p-values and Cohen’s d. All analyses were carried out in SPSS 24 (IBM Corporation, Somers, NY).

**Table 2:** Main and interaction effects from general linear repeated measures models.

	*MWT*					
Analysis 1	Group		Condition		Group × condition	
	F	p	F	p	F	p
Alpha ERSP (0–500 ms)	0.33	0.566	0.741	0.394	2.34	0.134
Alpha ERSP (500–1000 ms)	7.89	0.008*	22.15	0.001*	2.95	0.094
Beta ERSP (0–1000 ms)	6.94	0.012*	34.59	0.001*	0.440	0.511
Theta ERSP (0–500 ms)	12.67	0.001*	66.48	0.001*	6.95	0.012*
Theta ITC (0–500 ms)	20.29	0.001*	107.52	0.001*	4.89	0.032*

Analysis 2	Group		Probe		Group × probe	
	F	p	F	p	F	p

Alpha ERSP (0–500 ms)	5.70	0.022*	0.25	0.620	0.01	0.982
Alpha ERSP (500–1000 ms)	13.86	0.001*	0.15	0.701	0.37	0.548
Beta ERSP (0–1000 ms)	4.44	0.042*	1.64	0.208	0.32	0.577
Theta ERSP (0–500 ms)	5.33	0.027*	2.04	0.163	0.92	0.345
Theta ITC (0–500 ms)	11.32	0.002*	0.22	0.645	9.77	0.003*

	*SAT*					
	Group		Delay		Group × delay	
Analysis 1	F	p	F	p	F	p

Alpha ERSP (0–500 ms)	1.46	0.234	2.99	0.034*	0.452	0.716
Alpha ERSP (500–1000 ms)	7.63	0.008*	0.326	0.806	0.268	0.849
Beta ERSP (0–750 ms)	2.30	0.139	0.80	0.497	1.71	0.171
Beta ERSP (750–1500 ms)	10.80	0.002*	0.05	0.952	0.51	0.604
Theta ERSP (0–500 ms)	4.22	0.047*	18.40	0.001*	0.408	0.748
Theta ITC (0–500 ms)	0.07	0.793	0.674	0.522	1.15	0.322

Analysis 2	Group		Probe		Group × probe	
	F	p	F	p	F	p

Alpha ERSP (0–500 ms)	8.68	0.006*	0.67	0.418	1.33	0.257
Alpha ERSP (500–1000 ms)	7.38	0.010*	0.59	0.448	1.44	0.240
Beta ERSP (0–1000 ms)	1.30	0.263	0.51	0.482	0.44	0.512
Theta ERSP (0–500 ms)	6.62	0.015*	1.36	0.252	2.07	0.160
Theta ITC (0–500 ms)	9.05	0.005*	1.40	0.245	4.36	0.045*

Abbreviations: ERSP- event-related spectral perturbation, MWT- Mind Wandering task, SAT- Sustained Attention Task, ITC- inter-trial coherence.

Notes: *p < 0.05. General linear repeated measures models tested for main effects of group (ADHD vs controls), condition (in the MWT, 1-back vs 0-back), delay (in the SAT, 1 s, 2 s, 5 s, 8 s) or probe (MW vs task focus), and two-way interactions (group-by-condition, group-by-delay or group-by-probe) on ERSP measures.

**Table 3:** Comparisons between and within groups on ERSP measures during task conditions (Analysis 1).

Between-group comparisons					
		ADHD vs Control		ADHD vs Control (covarying MW)	
*MWT*		*d*	p	*d*	p
Alpha ERSP	*1back*	0.02	0.930	0.03	0.904
0–500 ms	*0back*	*0.59*	0.038‡	0.14	0.606
Alpha ERSP	*1back*	**0.80**	0.012*	0.33	0.227
500–1000 ms	*0back*	*0.70*	0.021‡	0.09	0.719
Beta ERSP	*1back*	*0.55*	0.046‡	0.24	0.359
0–1000 ms	*0back*	*0.71*	0.014*	0.43	0.096
Theta ERSP	*1back*	**1.11**	0.001*	*0.61*	0.024‡
0–500 ms	*0back*	**0.82**	0.005*	0.48	0.072
Theta ITC	*1back*	**1.30**	0.001*	*0.60*	0.024‡
0–500 ms	*0back*	**1.00**	0.002*	*0.72*	0.007*
*SAT*		***d***	**p**	***d***	**p**
Alpha ERSP	*1 s*	*0.60*	0.036‡	*0.62*	0.034‡
0–500 ms	*2 s*	0.29	0.325	0.27	0.279
	*5 s*	0.18	0.539	0.23	0.392
	*8 s*	0.20	0.511	0.22	0.343
Alpha ERSP	*1 s*	**0.83**	0.007*	*0.72*	0.010*
500–1000 ms	*2 s*	*0.79*	0.012*	0.49	0.051
	*5 s*	*0.73*	0.014*	*0.53*	0.041‡
	*8 s*	*0.60*	0.032‡	0.42	0.093
Beta ERSP	*2 s*	0.49	0.097	*0.70*	0.016‡
750–1500 ms	*5 s*	*0.56*	0.054	*0.55*	0.068
	*8 s*	*0.71*	0.020‡	*0.60*	0.033‡
Theta ERSP	*1 s*	**0.81**	0.006*	0.49	0.145
0–500 ms	*2 s*	0.32	0.267	0.21	0.422
	*5 s*	0.49	0.091	0.41	0.097
	*8 s*	0.33	0.259	0.05	0.929

Within-group comparisons									
		ADHD		ADHD (covarying MW)		Controls		Controls (covarying MW)	
*MWT*		*d*	p	*d*	p	*d*	p	*d*	p

Alpha ERSP 0–500 ms	*1back* vs *0back*	0.12	0.575	0.11	0.590	0.31	0.156	0.27	0.223
Alpha ERSP 500–1000 ms	*1back* vs *0back*	*0.60*	0.012‡	0.59	0.013‡	**0.82**	0.001*	*0.73*	0.003*
Beta ERSP	*1back* vs *0back*	**0.80**	0.001*	**0.80**	0.001*	**0.90**	0.001*	**0.84**	0.001*
Theta ERSP	*1back* vs *0back*	*0.73*	0.003*	**0.80**	0.002*	**1.71**	0.001*	**1.58**	0.001*
Theta ITC	*1back* vs *0back*	**1.18**	0.001*	**1.58**	0.001*	**1.70**	0.001*	**1.91**	0.001*

*SAT*		*d*	p	*d*	p	*d*	p	*d*	p

Alpha ERSP	*2 s* vs *5 s*	0.49	0.035‡	*0.61*	0.025‡	0.25	0.225	0.31	0.134
0–500 ms	*2 s* vs *8 s*	*0.70*	0.003*	*0.78*	0.002*	0.27	0.174	0.29	0.143
	*5 s* vs *8 s*	0.02	0.925	0.02	0.928	0.04	0.828	0.04	0.837
Alpha ERSP	*2 s* vs *5 s*	0.12	0.582	0.13	0.541	0.09	0.628	0.13	0.520
500–1000 ms	*2 s* vs *8 s*	0.11	0.603	0.08	0.717	0.06	0.773	0.07	0.705
	*5 s* vs *8 s*	0.01	0.962	0.07	0.745	0.01	0.975	0.01	0.992
Beta ERSP	*2 s* vs *5 s*	0.03	0.893	0.09	0.688	0.08	0.684	0.05	0.801
750–1500 ms	*2 s* vs *8 s*	0.13	0.541	0.16	0.471	0.23	0.285	0.16	0.450
	*5 s* vs *8 s*	0.20	0.353	0.10	0.626	0.15	0.469	0.12	0.564
Theta ERSP	*2 s* vs *5 s*	0.05	0.816	0.03	0.885	0.15	0.436	0.23	0.246
	*2 s* vs *8 s*	0.42	0.052	0.42	0.054	0.46	0.029‡	0.47	0.026‡
	*5 s* vs *8 s*	0.36	0.095	0.33	0.122	0.48	0.024‡	0.42	0.051

Abbbreviations: MWT- Mind Wandering task, SAT- Sustained Attention Task, MW – Mind Wandering.

Notes: *FDR correction significant at p ≤ 0.014, ‡trend-level effects at p ≤ 0.05. **Bold**: d≥0.80 indicating large effect size, *Italics*: d≥0.50 indicating a medium effect size. Only variables that showed significant effects in Table 2 were followed up in the post-hoc analysis testing between- and within-group effects.

We have also conducted sensitivity analyses to ensure group differences were not driven by participants with ADHD taking stimulant and non-stimulant medication. We compared controls and individuals with ADHD, who have not been treated with stimulants and/or non-stimulants, on ERSP and ITC measures. The sensitivity analyses provided similar results (i.e., effect sizes and significance values) to Analysis 1 and 2 (Supplementary Table 4, Supplementary Table 5).

**Table 4:** Comparisons between and within groups for all ERSP measures during periods of MW and task focus (Analysis 2).

Between-group comparisons			
		ADHD vs Controls	
*MWT*		*d*	p
Alpha ERSP	Task focus	*0.69*	0.025‡
0–500 ms	MW	*0.60*	0.062
Alpha ERSP	Task focus	**1.14**	0.001*
500–1000 ms	MW	*0.67*	0.035‡
Beta ERSP	Task focus	**0.85**	0.007*
0–1000 ms	MW	0.39	0.271
Theta ERSP	Task focus	*0.77*	0.024‡
0–500 ms	MW	0.48	0.129
Theta ITC	Task focus	0.38	0.228
0–500 ms	MW	**1.26**	0.001*
*SAT*		***d***	**p**
Alpha ERSP	Task focus	*0.61*	0.042‡
0–500 ms	MW	**1.02**	0.006*
Alpha ERSP	Task focus	*0.52*	0.066
500–1000 ms	MW	**1.07**	0.001*
Theta ERSP	Task focus	*0.61*	0.044‡
0–500 ms	MW	*0.63*	0.055
Theta ITC	Task focus	0.16	0.645
0–500 ms	MW	*0.74*	0.023‡

Within-group comparisons					
		ADHD		Controls	
*MWT*		d	p	d	p

Alpha ERSP					
0–500 ms	Task focus vs MW	0.12	0.570	0.05	0.804
Alpha ERSP					
500–1000 ms	Task focus vs MW	0.06	0.789	0.11	0.602
Beta ERSP	Task focus vs MW	0.26	0.232	0.12	0.586
Theta ERSP	Task focus vs MW	0.07	0.742	0.36	0.112
Theta ITC	Task focus vs MW	0.39	0.086	0.47	0.036‡
*SAT*		**d**	**p**	**d**	**p**
Alpha ERSP					
0–500 ms	Task focus vs MW	0.05	0.797	0.33	0.204
Alpha ERSP					
500–1000 ms	Task focus vs MW	0.07	0.761	0.35	0.174
Theta ERSP	Task focus vs MW	0.05	0.819	0.34	0.152
Theta ITC	Task focus vs MW	0.16	0.478	0.43	0.051

Abbreviations: MWT- Mind Wandering task, SAT- Sustained Attention Task, MW- Mind Wandering Episodes, MRT- Mean Reaction Time, RTV- Reaction Time Variability.

Notes: *FDR correction significant at p ≤ 0.007, ‡trend-level effects at p < 0.05. **Bold**: d≥0.80 indicating large effect size, *Italics*: d≥0.50 indicating a medium effect size. Analyses 2 included 21 controls and 21 individuals with ADHD in the MWT, and 18 controls and 23 individuals with ADHD in the SAT.

## 5 Results

All main and interaction effects are displayed in Table 2. In this section, we focus on between- and within-group post-hoc comparisons.

### 5.1 Analysis 1: Low vs high demand (Table 3)

#### 5.1.1 Between-group comparisons

Compared to controls, adults with ADHD showed significantly weaker alpha (Fig. 1.A, Fig. 2.A) and beta (Fig. 1.B, Fig. 2.B) decreases across demands in both tasks, except for alpha during 0-back and beta during 1-back and 8 s delays, which were trend-level effects. Individuals with ADHD also showed weaker theta power increase (Fig. 1.C) and theta phase consistency (Fig. 1.D) during high (1-back) and low (0-back) demand on working memory (MWT), but not during high or low demand on sustained attention (SAT, Fig. 2.B).

#### 5.1.2 Within-group comparisons

Both groups showed significantly weaker beta decrease (Fig. 1.B), theta increase (Fig. 1.C) and theta phase consistency (Fig. 1.D) during the 0-back compared to the 1-back condition in the MWT (0–1000 ms), and no differences in beta power (Fig. 2.B) and theta phase consistency between delays in the SAT. Controls also showed weaker alpha during 0-back compared to the 1-back condition (Fig. 1.A), whereas this effect was at trend level in the ADHD group. By contrast, individuals with ADHD, but not controls, showed significantly weaker alpha decrease during the 2 s compared to the 8 s, and the 5 s delays at trend level, following stimulus presentation (0–500 ms) (Fig. 2.A). There were trend-level effects of higher theta increase during 5 s and 8 s delays compared to the 2 s delays in both groups (Fig. 2.B).

#### 5.1.3 MW frequency as a covariate

After controlling for MW, most differences between ADHD and control groups for alpha, beta, and theta ERSP became either a trend or non-significant across both tasks, and the effect sizes became small (Table 3). Exceptions were beta ERSP during the 8 s delays, where the group difference remained a trend, and the group effects for 2 s, which was not significant and became a trend after controlling for MW. The between-group effects for theta ITC remained significant in the 0-back and at trend-level in the 1-back, although the effect sizes were reduced from large to medium. The within-group effects for all variables remained unchanged in both groups after adding MW as a covariate (Table 3).

### 5.2 Analysis 2: MW vs task focus (Table 4)

#### 5.2.1 Between-group comparisons

During task focus, significant or trend-level effects indicated weaker alpha decrease (Fig. 3.A) and weaker theta increase (Fig. 3.B) across tasks, as well as weaker beta decrease in the MWT during task focus (Supplementary Fig. 3), in adults with ADHD compared to controls. During MW episodes, the ADHD group showed significantly weaker alpha decrease in the SAT (Fig. 4.A) and lower theta ITC (Fig. 3.C) in the MWT, with further trend-level effects for alpha decrease in the MWT (Fig. 3.A) and theta ITC in the SAT (Fig. 4.C). Beta power (Supplementary Fig. 3) showed non-significant main or interaction effects in the SAT (Table 2), therefore post-hoc tests were not run for this measure.

#### 5.2.2 Within-group comparisons

Both groups did not show within-group differences between periods of task focus and MW in alpha (Fig. 3, Fig. 4.A), beta (Supplementary Fig. 3), or theta (Fig. 3, Fig. 4.B) power, nor theta ITC (Fig. 3, Fig. 4.C) during either task.

## 6 Discussion

To the best of our knowledge, this is the first study to investigate event-related changes of brain oscillatory activity with changing cognitive demands and across episodes of MW and task focus in an ADHD sample, using an experience-sampling approach for measuring MW. Consistent with our hypotheses, adults with ADHD showed alterations in event-related oscillations associated with reduced inhibition of task-irrelevant information during high working memory demands and across low and high sustained attention demands. The ADHD group further showed lower attention allocation and more variable stimulus processing across working low and high memory demands and during low sustained attention demands, as well as impaired response execution during low working memory demands. These group differences were partly explained by greater MW frequency in the ADHD group, suggesting that MW may play a role in the atypical EEG patterns displayed by individuals with ADHD. During task focus, adults with ADHD compared to controls showed impaired response execution and inhibition of task-irrelevant information, specifically during the MWT. Instead, during MW periods, the ADHD group showed lower consistency of stimulus processing in the MWT and weaker inhibition of task-irrelevant information in the SAT relative to controls. These findings suggest that atypical EEG profiles associated with reduced inhibition of task-irrelevant information and more variable stimulus processing are implicated in increased spontaneous MW in adults with ADHD. These MW-related EEG patterns may represent promising neural markers that, in the future, could be used for real-time monitoring of treatment effects in adults with ADHD.

Our first aim was to compare individuals with and without ADHD on event-related oscillatory patterns during task conditions eliciting varying levels of MW (Analysis 1). Adults with ADHD compared to controls showed weaker alpha power decreases during high demand on working memory and during both high and low demand on sustained attention, reflecting reduced inhibition of task-irrelevant information. These effects in alpha modulations in both tasks extend replicated oscillatory findings in ADHD samples (Michelini et al., 2022) and support current views suggesting that atypical alpha is a neural correlate of ADHD-related attentional difficulties (Lenartowicz et al., 2018). Adults with ADHD also showed lower theta power increase and theta phase consistency than controls across working memory demands, but no difference during varying sustained attention demands. This is consistent with the role of theta power in working memory processes (Jensen and Tesche, 2002, Hsieh and Ranganath, 2014) and working memory deficits in ADHD (Lenartowicz et al., 2014, Michelini et al., 2022). The lower theta phase consistency in the ADHD group further aligns with evidence that the disorder is associated with neural inefficiency, particularly during cognitive challenging tasks like the MWT (Groom et al., 2010). Conversely, varying sustained attention demands might elicit more optimal levels of attention allocation, as both groups showed comparable theta power across delays (from 2 s to 8 s), extending previous findings showing no group differences on P3 across sustained attention demands (Bozhilova et al., 2021a, Bozhilova et al., 2021b). The ADHD group further showed lower theta power than controls during high demand on sensorimotor function (1 s) in the SAT, suggesting that frequent and predictable stimuli might elicit neural activity associated with higher MW frequency due to their high automaticity. Finally, adults with ADHD displayed weaker beta power decrease than controls during low working memory demands, suggesting impaired motor response execution processes. Together, these findings indicate that conditions characterised by low cognitive demands and associated with higher MW frequency (Bozhilova et al., 2021a, b) tend to elicit particularly pronounced impairments in brain activity in adults with ADHD. Treatment approaches targeting MW frequency and associated brain patterns might thus be promising for individuals with ADHD.

In analyses examining within-group adjustments in brain oscillations with changing cognitive demands, we found a general pattern of improvements in oscillatory activity from low to high demand on working memory in both groups, evidence by stronger beta decrease, theta increase and theta phase consistency. While controls showed significant improvement in alpha activity from low to high working memory demands, this difference was not significant in the ADHD group, potentially suggesting lower ability to suppress task-irrelevant information in response to increasing cognitive demands. In the SAT, controls maintained consistent inhibition of task-irrelevant information across cognitive demands, as reflected by comparable alpha decreases across delays, the ADHD group showed weaker alpha decreases during low compared to high sustained attention demands. Together, these results extend our previous findings pointing to context regulation of MW frequency and associated ERPs across groups in the MWT, and in controls – but not adults with ADHD – in the SAT (Bozhilova et al., 2021a, Bozhilova et al., 2021b).

MW frequency statistically explained most of the group differences alpha power decreases during varying demands on working memory and sustained attention. Nevertheless, as the group difference on alpha decrease during low and high sustained attentions demand did not reach statistical significance, further research in larger samples is needed to confirm these findings. Group differences in response execution (beta decrease), attention allocation (theta increase) and variability of stimulus processing (theta phase consistency) were only partly explained by MW frequency, suggesting that processes independent of MW and related to task demands may also play a role in group differences in these EEG patterns. Conversely, MW frequency did not statistically explain any of the within-group effects, suggesting a more limited effect of MW on adaptations to changing cognitive demands.

Our second aim was to assess the relationship between MW and oscillatory activity more directly by contrasting periods of MW and task focus in individuals with and without ADHD (Analysis 2). Adults with ADHD showed lower inhibition of task-irrelevant information (i.e., weaker alpha decreases) compared to controls during task focus in the MWT and during MW in the SAT, with similar patterns not reaching statistical significance during MW in the MWT and during task focus in the SAT. These findings suggest that atypical EEG patterns reflecting difficulties inhibiting task-irrelevant information in ADHD may be particularly pronounced during periods of task focus in cognitively challenging tasks like the MWT, as well as during MW in less challenging tasks like the SAT. By contrast, the lack of group differences in alpha power during MW in the MWT suggest that individuals with and without ADHD have comparable suppression of task-irrelevant information during MW in this challenging task, consistent with well-established models of MW (Christoff, Irving, Fox, Spreng, & Andrews-Hanna, 2016). Adults with ADHD also showed greater variability of stimulus processing (i.e., weaker theta phase consistency) than controls during MW in the MWT (with a similar pattern not reaching statistical significance in the SAT), but not during task focus. As such, decreased neural efficiency in ADHD may be specific to MW periods, consistent with a link between theta phase consistency and attentional impairments in individuals with ADHD (Vainieri et al., 2020). Compared to controls, adults with ADHD also showed significantly weaker beta increases during task focus in the MWT, but no differences during MW nor task focus in the SAT, consistent with the group differences in response execution during the more challenging MWT identified in Analysis 1. Together, these findings suggest that EEG patterns associated with reduced inhibition of task-irrelevant information and inconsistent stimulus processing underlie MW episodes in adults with ADHD.

## 7 Limitations and future directions

First, the small sample size did not allow for an exploration of more subtle effects (*d* < 0.50) and likely resulted in non-significant effects, such as non-significant interactions. Second, in the analyses comparing periods of MW and task focus, some participants had an insufficient number of trials and had to be excluded. This might explain why we did not find the hypothesised within-group differences in EEG activity between MW and task focus. Future research should replicate these findings in larger samples, perhaps using paradigms that induce a greater proportion of MW episodes, for example by manipulating the task difficulty. Future studies could also consider using other methods for detecting the onset of MW and its fluctuations during the task, such as pupil diameter (Pelagatti et al., 2020) to preserve the natural flow of MW.

## 8 Conclusions

To the best of our knowledge, this is the first study to identify event-related brain oscillatory patterns associated with MW and task focus in individuals with ADHD. Alpha decrease and theta phase consistency distinguished between ADHD and control groups in task conditions eliciting high cognitive demands and associated with MW, as well as during MW episodes identified through a rigorous experience-sampling approach. These EEG patterns may thus reflect key neural mechanisms of increased MW frequency in ADHD. Since MW shows strong associations with ADHD symptoms and daily life difficulties, future studies should test whether these neural markers can be used to monitor the effects of treatments aiming to regulate MW in adults with ADHD, such as meditation techniques (Brandmeyer and Delorme, 2018, Lee et al., 2018).

## Declaration of Competing Interest

Professor Jonna Kuntsi has given talks at educational events sponsored by Medice: all funds are received by King’s College London and used for studies of ADHD. Professor Philip Asherson has received honoraria for consultancy to Shire/Takeda, Flynn-Pharma, Eli-Lilly, Janssen, Novartis, Lundbeck and Medice; educational/research awards from Janssen, Shire, Lilly, Novartis, Flynn Pharma, Vifor Pharma, GW Pharma and QbTech; speaker at sponsored events for Shire/Takeda, Lilly, Novartis, Medice, Janssen-Cilag and Flynn Pharma. Professor Katya Rubia has received a grant from Shire/Takeda for another project and consultancy fees from Lundbeck and Supernus pharmaceuticals which were received by King’s College London and used for studies of ADHD.
