# Supplementary material for: Event-related brain dynamics during mind wandering in attention-deficit/hyperactivity disorder: An experience-sampling approach
Source: Neuroimage Clin. 2022 Jun 1;35:103068. doi: 10.1016/j.nicl.2022.103068 (PMC9194650; doi:10.1016/j.nicl.2022.103068)
Supplement: Supplementary data 1 [file mmc1.docx]

**SUPPLEMENTARY MATERIALS**

*Task performance and MW frequency*

As reported in a previous study on this sample (Bozhilova, Kuntsi, Rubia, Michelini, & Asherson, 2021), adults with ADHD reported decreasing MW frequency during increasing demands on working (i.e., context regulation), but not on sustained attention (deficient context regulation) (Supplementary Table 1). Controls maintained continuous task focus across all conditions. Regarding task performance during task conditions, adults with ADHD compared to controls had slower and more variable responses during both high and low demand on working memory, and only during high sensorimotor demand/low demand on sustained attention (Supplementary Table 1). Compared to controls, adults with ADHD made more omission errors during both low and high demand on sustained attention. MW frequency explained all these between-group differences in task performance (Supplementary Table 1). Adults with ADHD compared to controls had more variable responses during MW, but not task focus in the MWT. Slower responses were observed in the ADHD group compared to the control group during both task focus and MW in the MWT. Across tasks, adults with ADHD made more errors than controls during MW, but not task focus. In the SAT, adults with ADHD showed slower and more variables responses during MW, but not task focus.

**Supplementary Table 1.** Group comparisons for behavioural measures

|  | | | **ADHD** | | **Control** | | ***d*** | **p** | | |
| --- | --- | --- | --- | --- | --- | --- | --- | --- | --- | --- |
|  | |  | | **Mean ± SD** | **Mean ± SD** |  | | |  |  |
| ***MWT*** | | | | | | | | | |  |
| MW Frequency | 1-back | | | 0.45±0.30 | 0.10±0.12 | **1.52** | | | <0.001*** |  |
|  | 0-back | | | 0.66±.0.21 | 0.16±0.19 | **2.45** | | | <0.001*** |  |
| MRT | 1-back | | | 1204.18±292.25 | 938.71±233.27 | **0.99** | | | 0.001*** |  |
|  | 0-back | | | 1105.05±356.92 | 824.64±215.73 | **0.95** | | | 0.001*** |  |
| RTV | 1-back | | | 544.41±141.04 | 341.67±139.83 | **1.41** | | | <0.001*** |  |
|  | 0-back | | | 468.38±254.55 | 288.33±172.56 | **0.83** | | | 0.004* |  |
| Errors | 1-back | | | 0.59±0.37 | 0.40±0.35 | *0.52* | | | 0.090 |  |
|  | 0-back | | | 0.30±0.27 | 0.36±0.32 | 0.20 | | | 0.480 |  |
| ***SAT*** | | | | | | | | | |  |
| MW Frequency | 2s | | | 0.53±0.32 | 0.13±0.19 | **1.52** | | | <0.001*** |  |
|  | 5s | | | 0.69±0.31 | 0.13±0.17 | **2.24** | | | <0.001*** |  |
|  | 8s | | | 0.68±0.34 | 0.18±0.19 | **1.83** | | | <0.001*** |  |
| MRT | 1s | | | 315.14±25.30 | 287.45±31.33 | **0.93** | | | 0.001*** |  |
|  | 2s | | | 379.00±29.18 | 370.48±37.76 | 0.23 | | | 0.381 |  |
|  | 5s | | | 395.70±25.71 | 378.01±33.30 | *0.55* | | | 0.050 |  |
|  | 8s | | | 406.23±27.69 | 379.49±34.29 | **0.81** | | | 0.003** |  |
| RTV | 1s | | | 67.61±9.84 | 55.45±9.10 | **1.24** | | | <0.001*** |  |
|  | 2s | | | 52.69±11.31 | 49.25±11.66 | 0.30 | | | 0.291 |  |
|  | 5s | | | 51.70±8.27 | 50.54±10.33 | 0.11 | | | 0.660 |  |
|  | 8s | | | 50.37±10.93 | 51.20±8.12 | 0.10 | | | 0.762 |  |
| Errors | 1s | | | 0.05±0.04 | 0.02±0.02 | **0.95** | | | <0.001*** |  |
|  | 2s | | | 0.06±0.04 | 0.04±0.03 | *0.56* | | | 0.050 |  |
|  | 5s | | | 0.08±0.05 | 0.05±0.04 | *0.65* | | | 0.010** |  |
|  | 8s | | | 0.09±0.04 | 0.06±0.04 | *0.73* | | | 0.020** |  |

Abbreviations: ADHD- Attention-deficit/hyperactivity disorder, MRT – mean reaction time, RTV- reaction time variability.

Notes: * significant at p≤0.032, **significant at p≤.05, ***significant at p≤.001, **Bold**: d≥.80 indicating large effect size, Italics: d≥.50 indicating a medium effect size, d≥.20 indicating a small effect size. Errors during the SAT have been calculated by dividing the total number of omission errors by the number of trials. These results have been reported in a previous publication (Bozhilova et al. 2021). MW frequency was calculated using the total number of MW episodes in each task condition divided by the total number of all episodes in each condition (task focus and MW).

**Supplementary Table 2.** Comparisons between and within groups for task performance during periods of MW and task focus

| **Between-group comparisons** | | | | | |
| --- | --- | --- | --- | --- | --- |
|  |  | **ADHD vs Controls** | | | |
| ***MWT*** |  | ***d*** | | **p** | |
| **MRT** | Task focus | **0.91** | | 0.002* | |
|  | MW | *0.68* | | 0.026‡ | |
| **RTV** | Task focus | 0.21 | | 0.457 | |
|  | MW | *0.72* | | 0.018* | |
| **Errors** | Task focus | 0.30 | | 0.297 | |
|  | MW | **1.11** | | 0.001* | |
| ***SAT*** |  | ***d*** | | **p** | |
| **MRT** | Task focus | **2.23** | | 0.001* | |
|  | MW | *0.51* | | 0.119 | |
| **RTV** | Task focus | **1.62** | | 0.001* | |
|  | MW | 0.32 | | 0.322 | |
| **Errors** | Task focus | *0.58* | | 0.056 | |
|  | MW | **1.15** | | 0.001* | |
| **Within-group comparisons** | | | | | |
|  |  | **ADHD** | | **Controls** | |
| ***MWT*** |  | **d** | **p** | **d** | **p** |
| **MRT** | Task focus vs MW | *0.61* | 0.006* | 0.39 | 0.075 |
| **RTV** | Task focus vs MW | *0.73* | 0.002* | 0.01 | 0.989 |
| **Error** | Task focus vs MW | *0.63* | 0.005* | 0.38 | 0.085 |
| ***SAT*** |  | **d** | **p** | **d** | **p** |
| **MRT** | Task focus vs MW | **1.17** | 0.001* | **1.17** | 0.001* |
| **RTV** | Task focus vs MW | **1.17** | 0.001* | **1.19** | 0.001* |
| **Errors** | Task focus vs MW | **0.88** | 0.001* | 0.25 | 0.302 |

Abbreviations: MWT- Mind Wandering task, SAT- Sustained Attention Task, MW- Mind Wandering Episodes, MRT- Mean Reaction Time, RTV- Reaction Time Variability

Notes: *FDR correction significant at p≤0.018, ‡trend-level effects at p<0.05. **Bold**: d≥.80 indicating large effect size, *Italics*: d≥.50 indicating a medium effect size. Analyses 2 included 21 controls and 21 individuals with ADHD in the MWT, and 18 controls and 23 individuals with ADHD in the SAT.

**Supplementary Table 3**. Comparisons between ADHD and control groups on the number of artefact free segments

|  | **ADHD**  **(N=23)** | **Controls**  **(N=25)** | **Group comparisons** | |
| --- | --- | --- | --- | --- |
|  | **Mean ± SD** | **Mean ± SD** | **d** | **p** |
| **0-back** | 37 ± 7 | 49 ± 10 | **1.35** | 0.001* |
| **1-back** | 169 ± 68 | 224 ± 56 | **0.87** | 0.003* |
| **1s** | 481 ± 166 | 634 ± 83 | **1.16** | 0.000* |
| **2s** | 35 ± 7 | 41 ± 7 | **0.84** | 0.007* |
| **5s** | 37 ± 8 | 46 ± 7 | **1.18** | 0.001* |
| **8s** | 37 ± 7 | 42 ± 8 | **0.65** | 0.027* |
| **MW~** | 88 ± 39 | 39 ± 15 | **1.63** | 0.001* |
| **TOT~** | 90 ± 20 | 158 ± 21 | **2.65** | 0.001* |

Abbreviations: ADHD- Attention-deficit/hyperactivity disorder, MW- Mind Wandering episodes, TOT- task focus episodes

Notes: * significant at p≤0.05, **Bold**: d≥.80 indicating large effect size, Italics: d≥.50 indicating a medium effect size, d≥.20 indicating a small effect size. ~This data is based on 21 controls and 21 individuals with ADHD due to the reduced number of MW episodes in controls.

**Supplementary Table 4.** Comparisons between groups on ERSP measures during task conditions.

| **Between-group comparisons** | | | | | | | | | |
| --- | --- | --- | --- | --- | --- | --- | --- | --- | --- |
|  |  | | | **ADHD vs Control** | | | **ADHD* vs Control** | |  |
| ***MWT*** | | | | ***d*** | | **p** | ***d*** | **p** | |
| **Alpha ERSP** | | | *1back* | 0.02 | | 0.930 | 0.08 | 0.811 |  |
| 0-500ms | | | *0back* | *0.59* | | 0.038‡ | *0.66* | 0.062 |  |
| **Alpha ERSP** | | | *1back* | **0.80** | | 0.012* | *0.76* | 0.048‡ |  |
| 500-1000ms | | | *0back* | *0.70* | | 0.021‡ | *0.70* | 0.069 |  |
| **Beta ERSP** | | | *1back* | *0.55* | | 0.046‡ | 0.48 | 0.158 |  |
| 0-1000ms | | | *0back* | *0.71* | | 0.014* | **1.07** | 0.002* |  |
| **Theta ERSP** | | | *1back* | **1.11** | | 0.001* | **1.25** | 0.002* |  |
| 0-500ms | | | *0back* | **0.82** | | 0.005* | **0.83** | 0.033‡ |  |
| **Theta ITC** | | | *1back* | **1.30** | | 0.001* | **1.32** | 0.001* |  |
| 0-500ms | | | *0back* | **1.00** | | 0.002* | **0.98** | 0.009* |  |
| ***SAT*** | | |  | ***d*** | | **p** | ***d*** | **p** |  |
| **Alpha ERSP** | | *1s* | | | *0.60* | 0.036‡ | *0.77* | 0.048‡ |  |
| 0-500ms | | *2s* | | | 0.29 | 0.325 | 0.29 | 0.444 |  |
|  | | *5s* | | | 0.18 | 0.539 | 0.26 | 0.497 |  |
|  | | *8s* | | | 0.20 | 0.511 | 0.29 | 0.447 |  |
| **Alpha ERSP** | | *1s* | | | **0.83** | 0.007* | **0.90** | 0.021‡ |  |
| 500-1000ms | | *2s* | | | *0.79* | 0.012* | *0.79* | 0.046‡ |  |
|  | | *5s* | | | *0.73* | 0.014* | **0.85** | 0.027‡ |  |
|  | | *8s* | | | *0.60* | 0.032‡ | *0.63* | 0.097 |  |
| **Beta ERSP** | | *2s* | | | 0.49 | 0.097 | 0.38 | 0.320 |  |
| 750-1500ms | | *5s* | | | *0.56* | 0.054 | 0.30 | 0.416 |  |
|  | | *8s* | | | *0.71* | 0.020‡ | *0.56* | 0.123 |  |
| **Theta ERSP** | | *1s* | | | **0.81** | 0.006* | **0.99** | 0.008* |  |
| 0-500ms | | *2s* | | | 0.32 | 0.267 | 0.48 | 0.186 |  |
|  | | *5s* | | | 0.49 | 0.091 | *0.63* | 0.067 |  |
|  | | *8s* | | | 0.33 | 0.259 | *0.53* | 0.128 |  |

Notes: * The second set of analysis includes only individuals with ADHD, who have not been treated with stimulant or non-stimulant medication for ADHD symptoms.

**Supplementary Table 5.** Comparisons between groups for all ERSP measures during MW and task focus.

| **Between-group comparisons** | | | | | | |
| --- | --- | --- | --- | --- | --- | --- |
|  |  | | **ADHD vs Controls ADHD* vs Controls** | | | |
| ***MWT*** |  | | ***d*** | **p** | ***d*** | **p** |
| **Alpha ERSP** | | Task focus | *0.69* | 0.025‡ | **0.87** | 0.022‡ |
| 0-500ms | | MW | *0.60* | 0.062 | *0.72* | 0.078 |
| **Alpha ERSP** | | Task focus | **1.14** | 0.001* | **1.13** | 0.005* |
| 500-1000ms | | MW | *0.67* | 0.035‡ | *0.54* | 0.166 |
| **Beta ERSP** | | Task focus | **0.85** | 0.007* | **1.12** | 0.003* |
| 0-1000ms | | MW | 0.39 | 0.271 | *0.56* | 0.131 |
| **Theta ERSP** | | Task focus | *0.77* | 0.024‡ | *0.65* | 0.094 |
| 0-500ms | | MW | 0.48 | 0.129 | 0.46 | 0.249 |
| **Theta ITC** | | Task focus | 0.38 | 0.228 | 0.47 | 0.292 |
| 0-500ms | | MW | **1.26** | 0.001* | **1.46** | 0.001* |
| ***SAT*** | |  | ***d*** | **p** |  |  |
| **Alpha ERSP** | | Task focus | *0.61* | 0.042‡ | **0.97** | 0.020‡ |
| 0-500ms | | MW | **1.02** | 0.006* | **1.22** | 0.005* |
| **Alpha ERSP** | | Task focus | *0.52* | 0.066 | 0.48 | 0.238 |
| 500-1000ms | | MW | **1.07** | 0.001* | **1.00** | 0.022‡ |
| **Theta ERSP** | | Task focus | *0.61* | 0.044‡ | **1.10** | 0.011* |
| 0-500ms | | MW | *0.63* | 0.055 | *0.67* | 0.107 |
| **Theta ITC** | | Task focus | 0.16 | 0.645 | 0.54 | 0.174 |
| 0-500ms | | MW | *0.74* | 0.023‡ | **0.88** | 0.027‡ |

Notes: * The second set of analysis includes only individuals with ADHD, who have not been treated with stimulant or non-stimulant medication for ADHD symptoms.


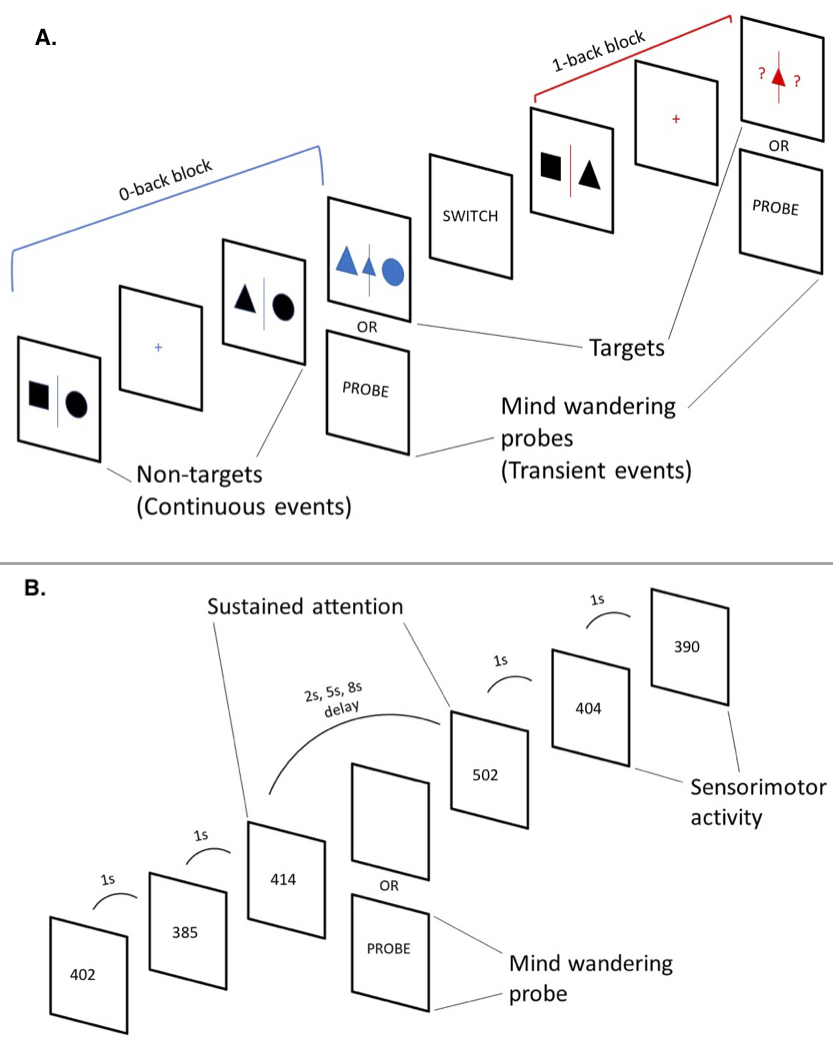


**Supplementary Figure 1.** *Schematic representation of the cognitive tasks*

**A. Mind Wandering Task:** The Participants alternated between the two conditions. One condition involved observing two black shapes (non-target) before three blue shapes (target) appeared. At that point, the participant had to indicate which of the two side shapes matches the small blue shape in the middle (choice reaction,0-back). In the 1-back condition, participants had to encode in working memory the two black shapes and when a small red shape with two red question marks on each side appears, they had to choose the left or right question mark based on the position of the black shape that is identical to the small red shape in the prior trial (working memory,1-back) (Konishi et al., 2015). **B. Sustained Attention task:** The participants were asked to respond as fast as possible to the appearance of black counters (participant’s reaction time) on the screen that count in milliseconds. The counters appeared either after frequent and predictable delays of 1 s in blocks of 3–5 stimuli, or after unpredictable long delays of 2,5 or 8 s, pseudo-randomly interspersed into the blocks of 1 s delays (Christakou et al., 2013).

**
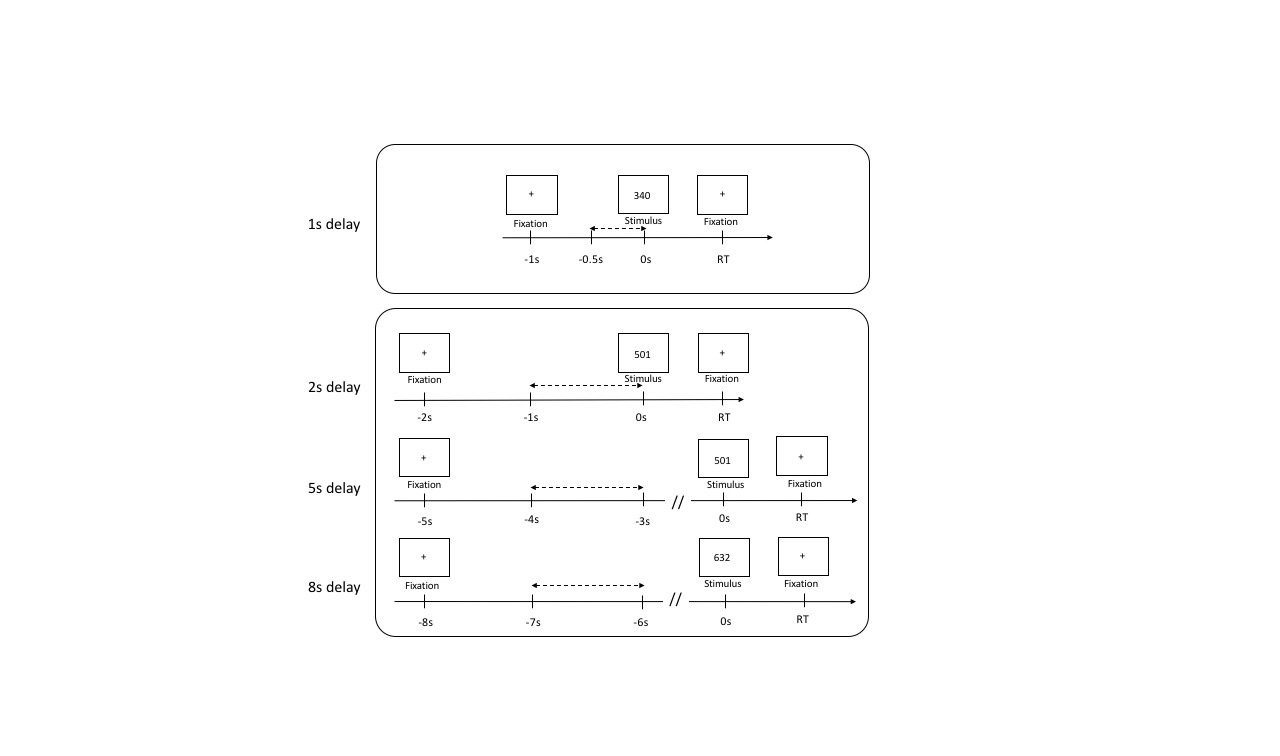
**

**Supplementary Figure 2.** *A schematic illustration of the temporal sequence of events in all delays (1s, 2s, 5s, 8s) of the Sustained Attention Task and of the pre-stimulus window used in time-frequency analyses.* All stimuli following each delay were followed by a response and the next stimuli appeared after 1s. The double-headed dashed window corresponds to the pre-stimulus baseline window (1s in the longer delays, and 0.5s in the shorter delay) used to normalize the event-related spectral perturbation (ERSP) and phase consistency (ITC) indices. In the 2s, 5s, and 8s delay conditions, we chose comparable pre-stimulus windows starting 1s after the fixation. We used a shorter pre-stimulus period in the 1s delay condition due to the shorter duration of the interval in this delay condition, whereby a longer pre-stimulus window (e.g., 1s) may have potentially overlapped with the response in the preceding trial. This difference in baseline correction did not affect the comparisons between conditions because the 1s delays were not included in the within-group analysis, which only involved 2s, 5s, and 5s delay conditions.


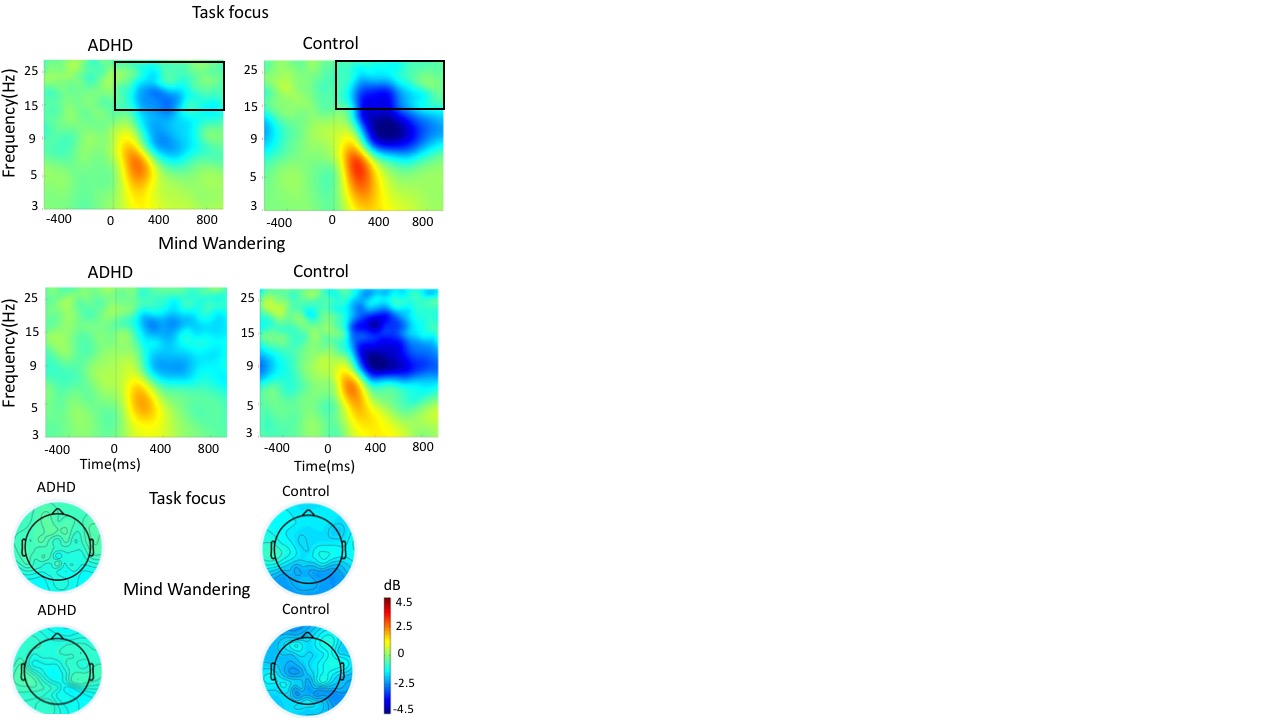


**Supplementary Figure 3.** Beta event-related spectral perturbation (ERSP) at centro-parietal regions in the ADHD and control groups during the MWT. ERSP plots during episodes of task focus and mind wandering (**Top**). Topographic maps by group in the 0–1000 ms window at task focus and mind wandering (**Bottom**).

**References:**

Bozhilova, N., Kuntsi, J., Rubia, K., Michelini, G., & Asherson, P. (2021). Electrophysiological modulation of sensory and attentional processes during mind wandering in attention- deficit/hyperactivity disorder. *NeuroImage: Clinical*, *29*, 102547.
